# Assessing Fish and Motile Fauna around Offshore Windfarms Using Stereo Baited Video

**DOI:** 10.1371/journal.pone.0149701

**Published:** 2016-03-02

**Authors:** Ross A. Griffin, Gary J. Robinson, Ashley West, Ian T. Gloyne-Phillips, Richard K. F. Unsworth

**Affiliations:** 1 Ocean Ecology Limited, Unit 5, Severnside Park, Epney, GL2 7LN, United Kingdom; 2 Seagrass Ecosystem Research Group, College of Science, Swansea University, Wallace Building, SA2 8PP, United Kingdom; 3 CMACS Ltd, 80 Eastham Village Road, Eastham, Wirral CH62 0AW, United Kingdom; Centro de Investigacion Cientifica y Educacion Superior de Ensenada, MEXICO

## Abstract

There remains limited knowledge of how offshore windfarm developments influence fish assemblages, particularly at a local scale around the turbine structures. Considering the existing levels of anthropogenic pressures on coastal fish populations it is becoming increasingly important for developers and environmental regulators to gain a more comprehensive understanding of the factors influencing fish assemblages. Improving our ability to assess such fish populations in close proximity to structures will assist in increasing this knowledge. In the present study we provide the first trial use of Baited Remote Underwater Stereo-Video systems (stereo BRUVs) for the quantification of motile fauna in close proximity to offshore wind turbines. The study was conducted in the Irish Sea and finds the technique to be a viable means of assessing the motile fauna of such environments. The present study found a mixture of species including bottom dwellers, motile crustaceans and large predatory fish. The majority of taxa observed were found to be immature individuals with few adult individuals recorded. The most abundant species were the angular crab (*Goneplax rhomboides*) and the small-spotted catshark (*Scyliorhinus canicula*). Of note in this study was the generally low abundance and diversity of taxa recorded across all samples, we hypothesise that this reflects the generally poor state of the local fauna of the Irish Sea. The faunal assemblages sampled in close proximity to turbines were observed to alter with increasing distance from the structure, species more characteristic of hard bottom environments were in abundance at the turbines (e.g. *Homarus gammarus*, *Cancer pagarus*, *Scyliorhinus spp*.) and those further away more characteristic of soft bottoms (e.g. Norwegian Lobster). This study highlights the need for the environmental impacts of offshore renewables on motile fauna to be assessed using targeted and appropriate tools. Stereo BRUVs provide one of those tools, but like the majority of methods for sampling marine biota, they have limitations. We conclude our paper by providing a discussion of the benefits and limitations of using this BRUV technique for assessing fauna within areas close to offshore windfarms.

## Introduction

Offshore windfarm development remains in its relative infancy. Although substantial strides have been made in terms of understanding the environmental impacts of such structures on biodiversity, there exists particular knowledge gaps in our understanding of the response of fish assemblages to these constructions [1–4]. Considering the existing levels of anthropogenic pressures on coastal fish populations [5], it is becoming increasingly important for developers and environmental regulators to gain a more comprehensive understanding of the factors influencing fish assemblages.

The introduction of structures associated with offshore renewable energy developments such as turbine foundations, scour protection and cable rock armour are thought to result in increases in local fish and shellfish populations. This is thought to be through the provision of new habitat, refuge and increased availability of food [6, 7]. Developing a detailed understanding of this new found habitat use requires the use of monitoring and assessment tools that are able to quantify fish and motile invertebrate assemblages effectively.

To date, fish assemblages at offshore wind farms have been assessed using conventional sampling techniques such as otter trawling, scientific beam trawling and deployment of gill/tangle nets [8–10]. The nature of these methods and their often destructive impact make them unsuitable for use within the close vicinity of turbine installations or on sensitive habitats. Assessments of fish assemblages associated with offshore structures are commonly included in Environmental Impact Assessments (EIAs) (in the UK) and any subsequent monitoring for offshore windfarm developments. In spite of such statutory requirements survey data usually relates to sampling undertaken ‘within the wind farm area’ rather than in close proximity to turbine foundations or other structures. This is because sampling is typically conducted using towed beam trawls [8].

Alternative non-destructive methods include Underwater Visual Census (UVCs) such as the use of Remotely Operated Vehicles (ROV) or SCUBA divers [11]. Although these techniques have previously been used at (or in close proximity to) offshore wind turbines, they are often expensive and in the case of diver surveys, carry significant health and safety risks so are therefore not routinely conducted. There are also numerous limitations and known biases with the use of UVCs such as observer bias and influence and the difficulties in making accurate length and hence biomass measurements [12, 13].

The use of stereo Baited Remote Underwater Video systems (BRUVs) provides a novel means of collecting robust and fit for purpose ecological data on motile fauna. The static and small nature of these systems means that they can potentially be applied to the survey of motile fauna in close proximity to offshore renewable structures. This technique has growing recognition for its use in quantifying fish and motile fauna in monitoring and assessment programmes across a variety of tropical and temperate habitats [11, 14–17]. It is non-destructive, repeatable and enables data to be collected on the relative abundances and size frequency distribution of motile fauna [18]. Importantly data can be collected with a high degree of accuracy [19].

Whilst single baited camera systems have been commonly used to enumerate fish in the low visibility waters typical of many sites in the North East Atlantic [20, 21] they are unable to provide accurate length measurements of subjects. Stereo BRUV systems overcome this limitation through the use of calibrated and synchronized pairs of cameras [19].

The use of a baited camera system does create an artefact of attracting species to a bait station. Various authors have investigated this effect relative to diver surveys and/or netting and found that bait increases abundance and species richness of generalist carnivores but does not influence herbivorous fish [22–24]. All forms of fish sampling are likely to incur some level of bias or misrepresentation with many techniques arguably semi-quantitative (at best). For example, most netting techniques are biased against fast swimming predators that are able to escape trawls [25, 26], static nets can be size selective [27] and biased against cryptic species or species of restricted mobility [28] whilst potting surveys are bias toward large individuals and may underestimate lobster and crab catch due to a high number of escapees [29]. Studies using BRUVs conducted in Atlantic seagrass meadows at sites where seine netting has also been used have shown lower species richness, with fewer cryptic species observed in seine net samples compared to BRUV data [15].

Stereo BRUV systems are commonly used in the tropics and throughout the southern hemisphere but their use in the North East Atlantic remains in its infancy. Studies in the UK have optimised established stereo BRUV methodologies for sampling fish and invertebrates in turbid coastal habitats [25, 30]. The use of stereo BRUV systems therefore presents a potentially viable sampling tool for assessing fish and motile invertebrates around offshore installations in the relatively low visibility waters of the North East Atlantic.

The present study examined the use of stereo BRUV systems for assessing fish and motile invertebrate fauna in close proximity to offshore windfarm structures. This study provides an assessment of the feasibility of the use of BRUV technology as an alternative non-destructive method of assessing relative abundance, diversity and age structure of fish and other motile fauna in temperate offshore environments within a renewable energy site, close to emplaced structures.

## Materials and Methods

### Study site

**Fig 1:**
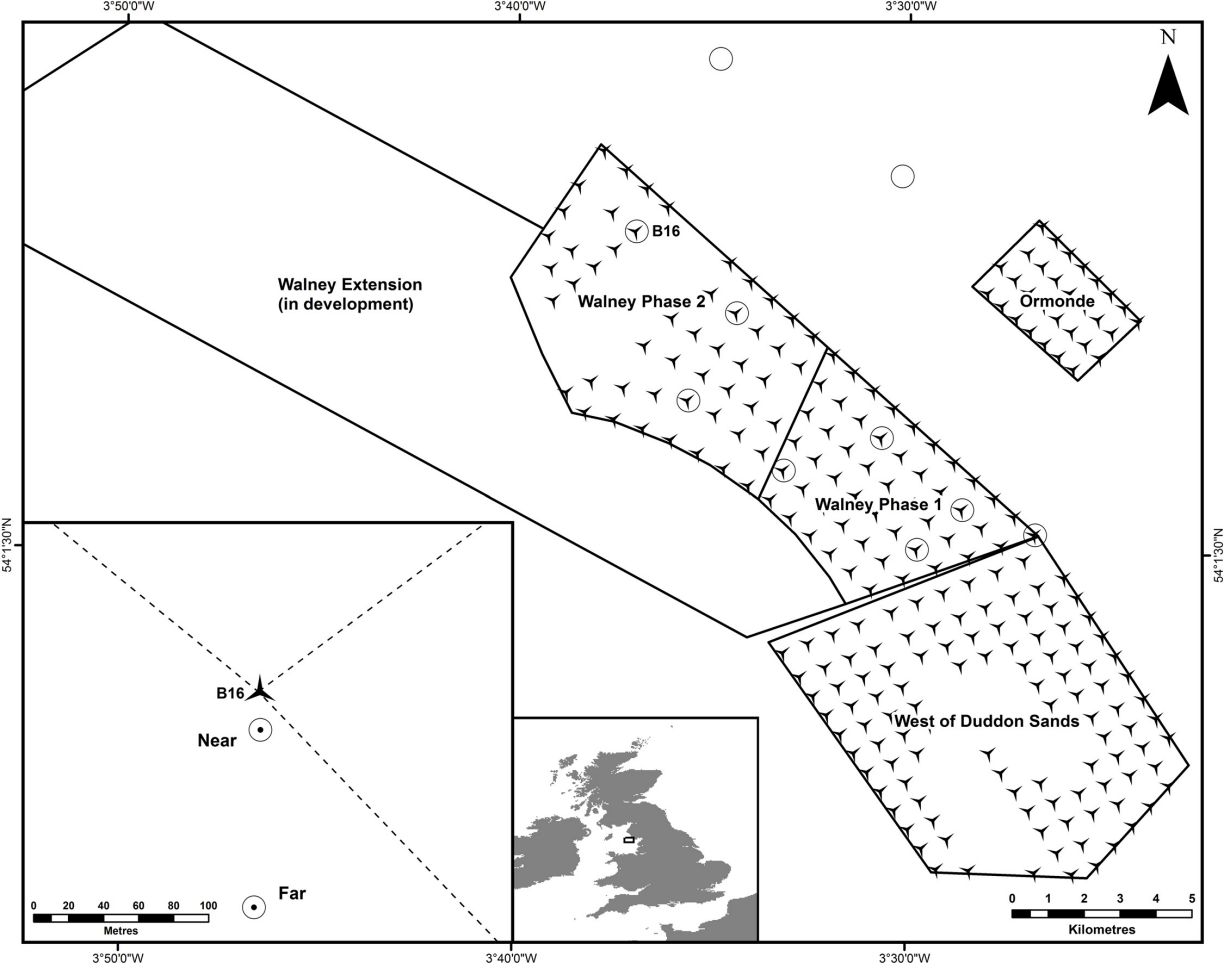
Location of the Walney Offshore Windfarm (WOWF) development and other adjacent offshore windfarms in the North East Irish Sea. The ten locations where twenty sites were sampled are shown by circles in the figure. Eight of these are located adjacent to wind turbines and two are located at a distance away to the north of Walney Phase 2. The inset figure (bottom left) shows the distance between the two sample sites placed at each location.

Motile fauna was sampled near and far from turbines at the Walney Offshore Windfarms (WOWF) between 25^th^ and 26^th^ July 2014. WOWF is located in the Irish Sea west of Walney Island (Fig 1). The WOWF comprises both the fully operational Walney Phase I and Phase II areas operated by Walney (UK) Offshore Windfarms Limited. It was commissioned in early 2012. The current study was undertaken with permission from DONG Energy, the owner of the offshore windfarms. Each wind turbine is supported by a steel monopile foundation of up to 6.5 m diameter at the seabed. The surrounding seabed is covered by a ring of approximately 20 m of rock armour to protect against sediment scour. Close sampling to each turbine relied on the use of a research vessel with dynamic positioning (supplied by Aquatech Ltd UK) and precise knowledge (using existing mapping data) of the outer edge of the rock armour at each location.

Benthic faunal community characterisation surveys conducted as part of the EIA (at turbines and reference sites) for the sites found all sites sampled in the present study to be broadly similar, containing no locations with particularly distinct communities [31]. The sediments were mostly dominated by mud and fine sand containing an abundance of annelids, small crustaceans and molluscs [31]. Given the construction of the turbines and the associated Rock Armour, those sites in close proximity to the turbines have additional rocky substrata that includes extensive crevices. Given this background information on the benthic habitat there is little evidence to suggest that large motile fauna between sites and distances (from turbines) should be influenced by anything other than the construction of wind turbines and associated Rock Armour. Environmental conditions (depth, salinity and exposure) were also similar across the sampling area.

### Experimental design

**Fig 2:**
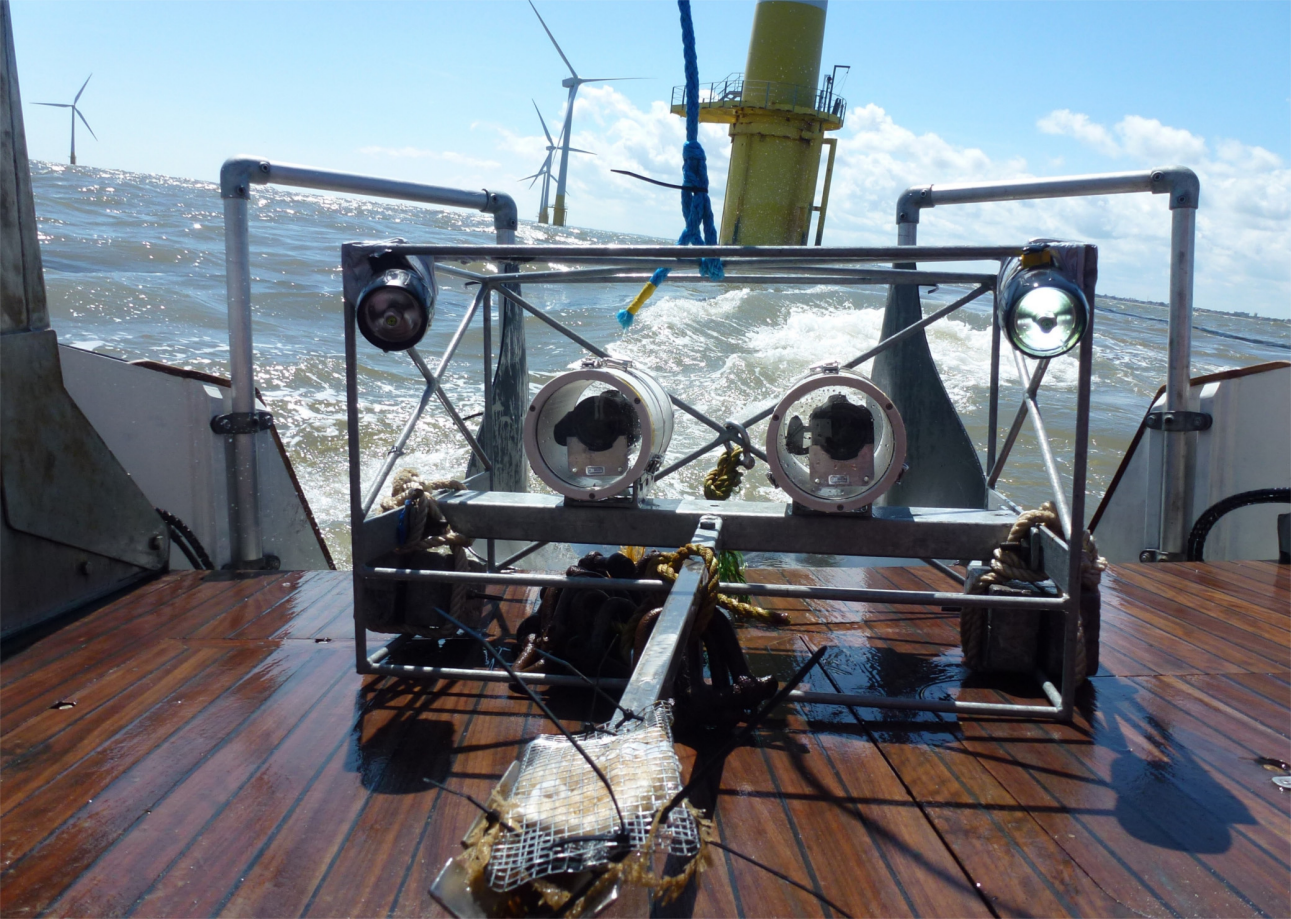
Stereo Baited Remote Underwater Video system being deployed in close proximity to an offshore wind turbine using a research vessel with dynamic positioning. Image shows stereo cameras placed alongside underwater dive lights and mounted within a steel cage. Bait bar and bait cage mounted to the front of the system. Additional lead weight is attached to base of the cage to ensure stability of system on the seafloor.

Species relative abundance, diversity and age structure of motile fauna was sampled within benthic habitats at locations of varying distance (100 m apart–near and far to the turbines) from eight turbine installations (at least 2 km apart from each other–see Fig 1) within the WOWF site (sixteen samples). Sampling was also conducted at two additional reference sites (a further 4 km from the turbines) resulting in a further four samples (Fig 1). Our study resulted in the collection of a total of twenty samples. Sampling used two stereo Baited Remote Underwater Video systems (stereo BRUVs) deployed during full daylight hours, sampling was spread evenly across the two days with no temporal bias between different distances, preventing any concerns with respect to diel influences on fish assemblages [32, 33]. These were placed on natural seabed (Fig 2). Stereo BRUV systems were deployed simultaneously (within 5 mins of each other) in a paired manner so that one system was near to the turbine (at the base of the Rock Armour) and one was far (at least 100 m away).

A distance of 100 m between the two paired samples was considered to provide a minimum point of independence from each other. Previous studies have used distances of 100 m to be independent [34], whilst others have used much larger distances (e.g. over 400 m) [35]. Although authors have attempted to justify such selections using estimates of current speed, fish density and home ranging behaviour, these values are largely wild guesses. Cappo et al. 2004 [35] developed an equation to model the influence of the bait plume. In our pilot study knowledge of the local current speeds and tidal movements was insufficient to use this equation at this time. Given the pilot status of this research in waters of very low fish density and visibility (comparatively to most previous BRUV studies), we have chosen to treat these samples (100 m apart) as independent. As with many such studies there is insufficient evidence to determine this with any level of certainty.

The deployment duration for each drop (a sample) was 1 hour. This duration was deemed suitable based on previous studies in the Irish Sea (species-time accumulation curves) with the same equipment and method [14, 15]. All deployments were in water depths of between 20 and 29.8 m and the surface Secchi disk readings ranged between 3.5 and 6 m.

### Equipment and data collection

The Swansea University stereo BRUV system used was a modified version of the SeaGIS equipment (See Fig 2) which is based on systems used in Australian research. The system consisted of two calibrated Canon high definition video cameras within PVC underwater housings, mounted at a fixed position on a galvanised steel frame with a 90 cm bait pole [15]. Bait comprised of oily fish has been found to be most effective in previous studies [36], therefore approximately 50 g of mackerel (*Scomber scombrus*) was used in each bait bag. 50g of bait has been found to be sufficient for the assessment of motile marine fauna using BRUVs in a range of other studies in the Irish Sea [14, 15] and the total weight of bait used has been found to have limited influence upon the assemblage structure of species attracted to BRUV systems [37]. Three underwater dive torches were also added to each system for improved illumination of the field of view (2 x Underwater Kinetics eLED Light Cannon and 1 x Underwater Kinetics SL3). This was due to the high turbidity and low light environment of the Irish Sea at depth [14, 15].

### Image analysis

All footage was analysed at Swansea University using the specialised SeaGIS software *EventMeasure* (Version 3.51) (www.seagis.com.au). This software was pre-calibrated using the SeaGIS software package *Cal* and cameras synchronised allowing for accurate length measurements of observed fish and motile fauna. The placement of a diode containing multiple flashing LED lights in front of the cameras prior to deployment enabled the cameras to be synchronised to the same video frame.

The footage was analysed using the left hand camera for identification of both new species and species relative abundance (*N*_*max*_). The right hand camera was used in tandem with the left hand camera to produce length measurements where the maximum species *N*_*max*_ was observed [38]. To ensure consistent precision of measurements, maximum RMS values in *Event Measure* were set at 10mm. All individual fish that were measured (total length) using stereo video were classified as being adult or juvenile relative to the size of maturation observed in the literature and recorded on FISHBASE [33]. Size of maturation data used for the species here are provided in Unsworth et al. 2014 [15]. Carapace length (CL) measurements were taken for crustaceans.

*N*_*max*_ is a metric commonly used for quantifying the relative abundance of fish observed on underwater video [38]. It counts the maximum number of fish recorded at any one time (single video frame) and therefore removes the concerns associated with potentially double counting individual fish [38]. Estimates of *N*_*max*_ are considered conservative, particularly in areas where fish occur in high densities [35, 39].

### Data analysis

All summary data is presented as means + or–standard deviation. Univariate one-way (unbalanced) ANOVA [40] was conducted in SigmaPlot v13 to test for any differences between the abundance and number of species present at each treatment (distance from turbine). ANOVA was conducted following data passing both the Shapiro-Wilk normality test and a Brown-Forsythe equal variance test [40]. Alternatively Kruskal-Wallis One Way Analysis of variance on ranks was used. All pairwise comparisons were performed using the Dunns method. Given the small sample size and the unbalanced nature of the experimental design only p-values <0.01 were considered to be of significance in order to reduce the risk of making a Type II error.

In order to determine the most efficient pre-treatment method prior to multivariate analyses, data were displayed as a shade plot with linear grey-scale intensity proportional to *N*_*max*_ values [41]. Species were clustered using the standard agglomerative method, based on the ‘index of association’ resemblances computed on species-standardised *N*_*max*_. The resulting dendrogram was rotated to maximise the seriation statistic *p*, non-parametrically correlating their resemblances on the distance structure of a linear sequence [42]. Analysis of differences in the structure of motile faunal assemblages between locations illustrated in the shade plot was conducted using multivariate non-metric multidimensional scaling ordination (nMDS) and Bray-Curtis cluster analysis using the software PRIMER v7 [42]. The Bray-Curtis similarity index was used to generate a rank similarity matrix, which was then converted into an MDS ordination. To check on the adequacy of the low-dimensional approximations seen in MDS the use of PRIMER v7 enabled clusters to be superimposed upon the MDS ordination [43]. An ordered one-way analysis of similarities (ANOSIM) was used to investigate differences identified from MDS and CLUSTER [42].

## Results

A total of 118 individuals (based on *N*_*max*_) from 14 separate taxa were identified in the 20 hours of BRUV footage collected during this study (see original data in S1 Dataset). Of the 14 taxa recorded, the majority were fish whilst 5 were mobile crustaceans (Table 1). This included the commercially important European lobster *(Homarus gammarus)*, Norway lobster (*Nephrops norvegicus)* and edible crab (*Cancer pagurus)*. Some individuals could not be identified to species level so were recorded at family level only (e.g. Paguridae, Triglidae). All data is supplied in our supporting information (S1 Dataset).

**Table 1:** Mean (± SD) relative abundance (*N*_*max*_) and length measurements (mm) for each taxa recorded using stereo BRUV at three different distances (adjacent, 100 m and 4 km) from turbines sampled at the Walney Offshore Windfarm (WOWF) development.

Family	Species	Common Name	*N*_*max*_ Adjacent		*N*_*max*_ 100m		*N*_*max*_ 4km		*N*_*max*_ All		Length All (mm)		
			Mean	SD	Mean	SD	Mean	SD	Mean	SD	Mean	Min	Max
Cyaneidae	*Cyanea capillata*	Lion's mane jellyfish	0.1	0.4	0.1	0.4	0	0	0.1	0.3	83	83	83
Cyaneidae	*Cyanea lamarckii*	Blue jellyfish	0.1	0.4	0	0	0	0	0.1	0.2	24	24	24
Nephropidae	*Homarus gammarus*	European lobster	1	0.8	0.4	0.5	0	0	0.6	0.7	63	39	81
Nephropidae	*Nephrops norvegicus*	Norway lobster	0.1	0.4	0.6	0.7	1	0	0.5	0.6	24	15	32
Paguridae	-	Hermit crab	0.5	0.8	0.1	0.4	0.3	0.5	0.3	0.6	-	-	-
Cancridae	*Cancer pagurus*	Edible crab	0.1	0.4	0	0	0	0	0.1	0.2	110	74	134
Goneplacidae	*Goneplax rhomboides*	Angular crab	0.5	1.1	1.3	1	2.8	1	1.3	1.3	20	12	29
Scyliorhinidae	*Scyliorhinus canicula*	Small-spotted catshark	1.1	0.6	1.3	0.5	0	0	1	0.7	400	301	479
Scyliorhinidae	*Scyliorhinus stellaris*	Nursehound	0.9	0.6	0.4	0.5	0	0	0.5	0.6	568	489	653
Gadidae	*Merlangius merlangus*	Whiting	0.5	0.8	1.3	0.9	1	0	0.9	0.8	110	59	193
Atherinidae	*Atherina presbyter*	Sand smelt	0.4	0.5	0.3	0.5	0.3	0.5	0.3	0.5	62	52	77
Triglidae	-	Gurnard	0.1	0.4	0	0	0	0	0.1	0.2	-	-	-
Labridae	*Ctenolabrus rupestris*	Goldsinny wrasse	0.1	0.4	0	0	0	0	0.1	0.2	-	-	-
Soleidae	-	Sole	0.3	0.5	0.4	0.5	0.5	0.6	0.4	0.5	74	44	104

Total relative abundance (*N*_*max*_) per sample ranged from 3 to 9 individuals (fish and motile invertebrates) and the number of taxa ranged from 3 to 7 in an individual sample. The average relative abundance per sample (*N*_*max*_) across all sites was 5.9 ± 0.4 SD. The most abundant taxa observed across all samples were the angular crab (*Goneplax rhomboides*) (1.3 ± 0.3), the small-spotted catshark (*Scyliorhinus canicula*) (1 ± 0.2) and the whiting (*Merlangius merlangus*) (0.9 ± 0.2) (see Table 1).

**Fig 3:**
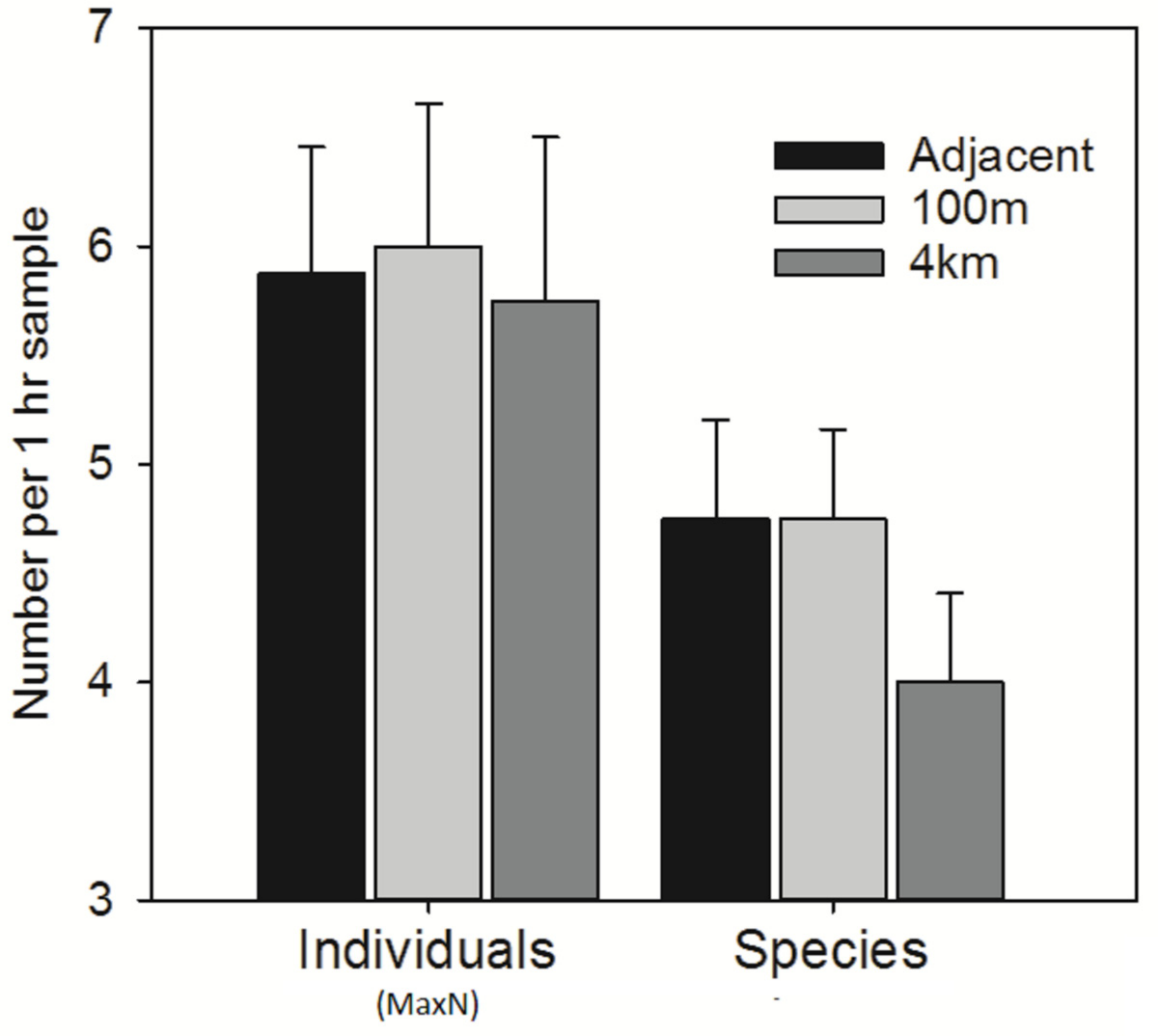
Mean (± SE) relative abundance (*N*_*max*_) and number of taxa of motile fauna recorded using stereo BRUV systems at three different distances (adjacent, 100m and 4km) from turbines sampled at the Walney Offshore Windfarm (WOWF) development. No significant differences were observed between any of these treatments.

**Fig 4:**
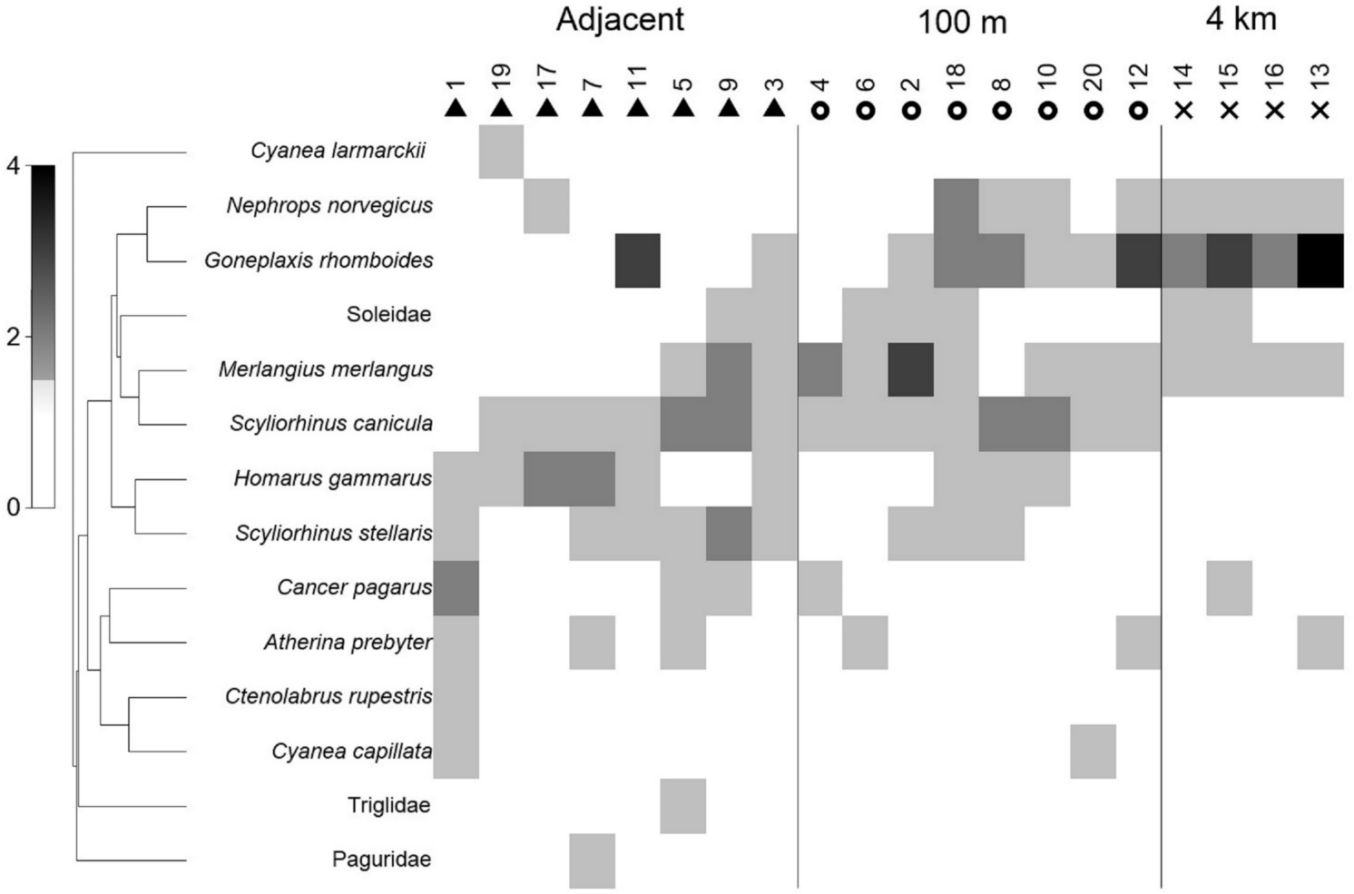
Shade plot of 14 taxa from 20 sites sampled with stereo BRUV systems at three different distances (adjacent, 100m and 4km) from turbines sampled at the Walney Offshore Windfarm (WOWF) development with linear grey-scale intensity proportional to untransformed *N*_*max*_ values. Species are clustered using the standard agglomerative method, based on the ‘index of association’ resemblances computed on species-standardised *N*_*max*_. The resulting dendogram is rotated to maximise the seriation statistic *p*, non-parametrically correlating their resemblances on the distance structure of a linear sequence [35].

**Fig 5:**
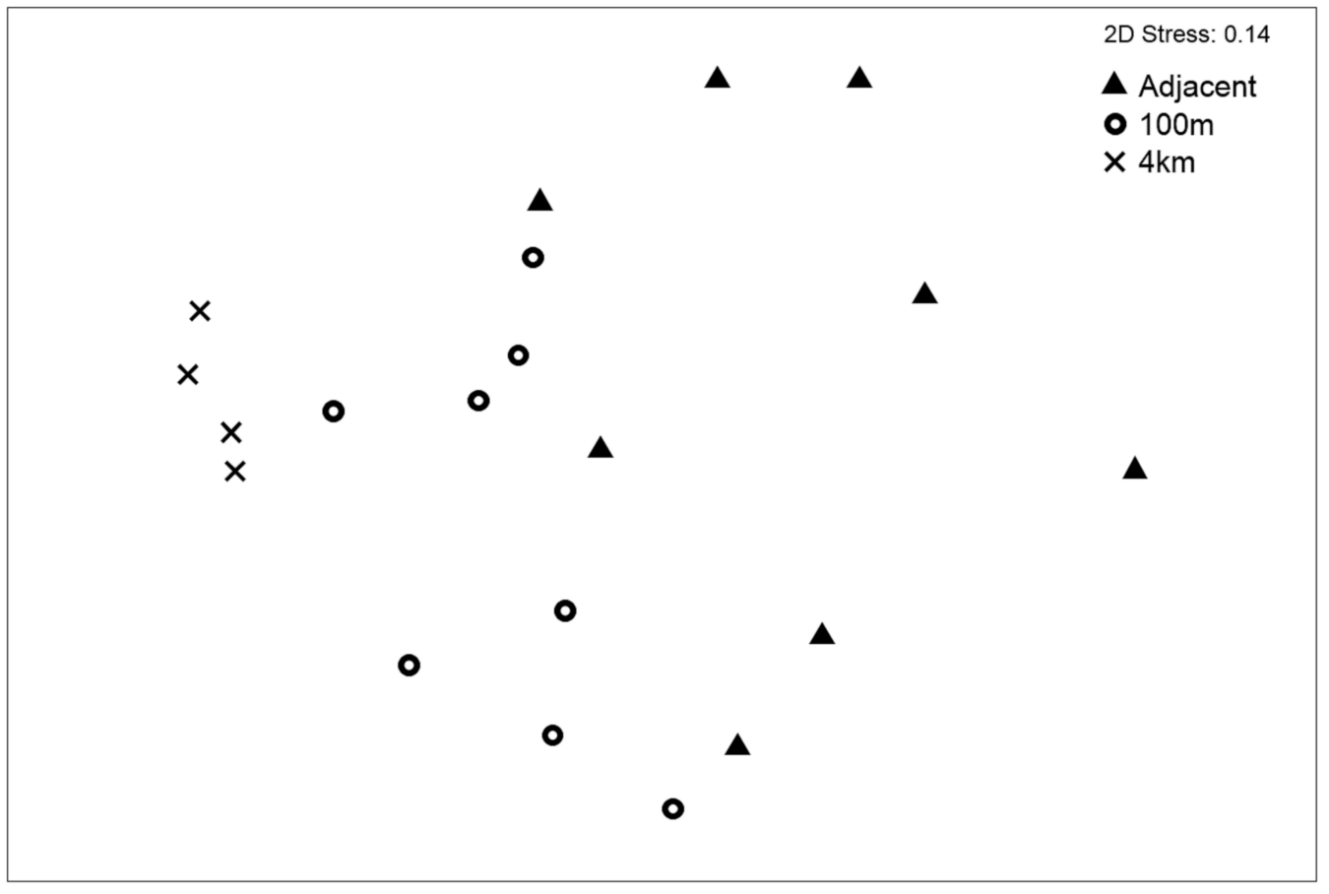
Non-metric multidimensional scaling ordination of community *N*_*max*_ data recorded using stereo BRUV systems at three different distances (adjacent, 100m and 4km) from turbines sampled at the Walney Offshore Windfarm (WOWF) development.

Faunal abundance did not vary significantly (ANOVA F_2,19_ = 0.03, P = 0.97) with increasing distance from the turbine (*N*_*max*_ adjacent: 6.00±1.85, *N*_*max*_ 100 m: 5.87±1.64, *N*_*max*_ 4 km: 5.75±1.50) (see Fig 3). The average number of taxa was slightly greater in samples taken adjacent and 100 m (adjacent: 4.75 ±1.16, 100m: 4.75 ±1.28) from the turbine installations than at the sites 4 km away (4.00 ±0.81), but these differences were again not significant (ANOVA F_2,19_ = 0.67, P>0. 53) (see Table 2). At the assemblage level, some significant differences were found (ANOSIM R = 0.43, P<0.05) (Fig 4). Pairwise analysis showsthese differences in the assemblage to exist between all distance pairs. The differences were most pronounced between the samples adjacent to the turbines and those 4 km from the windfarm site (ANOSIM R = 0.67, P = 0.002) (Table 2) demonstrated by the distinct separation of these points in Fig 5. The dissimilarity in the assemblages between the turbines and those 4 km from the windfarm site are driven by *Goneplaxis rhomboids*, *Scyliorhinus canicula*, *Homarus gammarus* and *Nephrops norvegicus* (SIMPER analysis).

**Table 2:** Summary statistics for ANOVA, Kruskal-Wallis and ANOSIM tests used to determine the presence of any differences in the relative density (*N*_*max*_) of abundant taxa and assemblage structure at three different distances (adjacent, 100 m and 4 km) from turbines sampled at the Walney Offshore Windfarm (WOWF) development. Where it was possible to conduct pairwise comparisons this data is also shown (* = P<0.05). H statistics are shown for Kruskal-Wallis (KW) tests, F statistics are presented from One-way ANOVA and R statistics presented for ANOSIM.

	DoF	H	R	F	P	Adj-100m	Adj-4km	100m-4km	Test Used
*N*_*max*_	2,19	n/a		0.03	nsd				ANOVA
No of taxa	2,19	n/a		0.67	nsd				ANOVA
Assemblage			0.43		p<0.05	nsd	*	*	ANOSIM
*Goneplaxis rhomboides*	2	8.06		n/a	P = 0.02	nsd	*	nsd	KW
*Scyliorhinus canicula*	2	10.04		n/a	p<0.01	nsd	*	*	KW
*Merlangius merlangus*	2	4.30		n/a	nsd				KW
*Homarus gammarus*	2	4.45		n/a	p<0.05	nsd	*	nsd	KW
*Scyliorhinus stellaris*	2	4.03		n/a	p<0.05	nsd	*	nsd	KW
*Nephrops norvegicus*	2	7.15		n/a	p<0.05	nsd	*	nsd	KW

Although average number of taxa recorded per sample did not change significantly with distance, total number of taxa did change. Fourteen different taxa were observed adjacent to the turbines, 10 taxa at 100 m from the turbines and only 6 taxa were recorded 4 km away. The low number at 4 km away should be treated with caution relative to the other distances as the sampling intensity was lower at that distance. The gurnard, the edible crab, the goldsinny wrasse and the blue jellyfish were all found adjacent to the turbines, but not at any other sites (see Table 1).

The relative abundance of the six most abundant species were examined relative to distance from turbine. *H*. *gammarus* were significantly more abundant (see Table 2) in samples taken adjacent to the turbines (1.0 ± 0.8) compared to samples taken 4 km from turbines (0.0 ± 0.0) and the converse was true for the Norwegian lobster (*Nephrops norvegicus*), with density of 0.1 ± 0.4 adjacent to the turbines and 1.0 ± 0.0 at 4 km from the turbines. The two species of Scyliorhinus were also significantly more abundant adjacent and 100 m to the turbines than at 4 km away (Table 2). Whiting (*Merlangius merlangus*) were not found to be significantly different in density with changes in distance from the turbines (Table 1).

Of the 14 taxa recorded it was possible to determine length measurements of 11 taxa. The inability to determine lengths of three of the taxa was due to either individuals not being observed concurrently on both stereo cameras (gurnard and goldsinny wrasse (*Ctenolabrus rupestris*) or the individual not showing features sufficient for measurement purposes (hermit crabs, (Paguridae)).

Most fish length ranges were relatively low in comparison to their known maximum lengths with all individuals being below the size at maturation estimates for each species [44]. The exception to this were the Scyliorhinidae with most individuals being sexually mature adults.

## Discussion

Much discussion has focussed on the potential use of offshore windfarms for enhancing biodiversity, and the areas around them for providing *de facto* marine reserves [1], but as the review by Ashley *et al*. (2014) reveals, there is a dearth of data investigating such effects, particularly within the peer reviewed literature and limited examination of the methods suitable to collect such data. Where data does exist it is largely collected at a distance from the turbines using extractive methods such as beam trawling. Here we evidence the viable use of stereo BRUV systems as a non-destructive means of assessing fish and motile invertebrate fauna in very close proximity to offshore windfarm structures.

The majority of comparable studies on the motile fauna of offshore wind turbines are from the Baltic Sea and northern part of the North Sea and provide little consideration for the inherent bias of fish sampling methodology. The methodology utilised to make such assessments is important given that studies around turbines in Sweden have found that small slender bottom dwelling fish are rarely caught in nets and larger fish were not seen in visual transects [45]. Such information backs up a plethora of data on fish sampling biases [18, 24, 27].

The present study found a mixture of species including bottom dwellers, motile crustaceans and large predatory fish. Although these were similar species assemblages to other studies published in the academic literature, the assemblages recorded in the present study were less diverse [7, 46]. Whether this is a local effect or the bias associated with the methodology remains to be seen but highlights the value in utilising multiple methods in order to be able to eliminate such possible explanations. Particular differences are the high abundance of gobies observed in other studies [7, 46].

Of note in this study was the generally low abundance and diversity measured across all samples. The low abundances of individuals potentially limited the statistical power to most clearly determine the effect of the turbines and underlines the need for high numbers of replicates in order to assess these environments effectively with a BRUV system. This may have been attributable to the low water clarity observed (2-3m) [35] and poor status of the fish populations of the Irish Sea [47, 48]. However, other recent studies with exactly the same equipment in the southern half of the Irish Sea have been in low visibility coastal habitats and have recorded higher fish densities [14, 15].

The present study found data to support the notion that community composition of motile fauna changed with distance from the turbines. These findings can however only be considered to be of a preliminary nature and need to be considered with caution due to the limitations of the present dataset. Species more commonly associated with hard bottom environments such as *H*. *gammarus*, *C*. *pagarus* and two species of catshark (*Scyliorhinus* spp.) were in higher relative abundance in close proximity to the turbines, whilst species commonly associated to soft bottom environments (e.g. Norwegian lobster and the angular crab) were more abundant outside the windfarm. Angular crab, in particular, were notably less abundant within the turbine array, possibly as a response to higher numbers of catshark that are known to feed opportunistically on a variety of crustacean prey in the Irish Sea [49]. Many of the taxa recorded in the present study are of commercial fisheries importance.

In conclusion, we find that stereo BRUV systems are a viable tool for assessing fish and other motile fauna in the vicinity of offshore windfarms and provide a means to elucidate differences in community composition related to the presence of the turbine structures and associated rock armour. This technology in tandem with appropriate surface support can be deployed safely in very close proximity to turbine structures and provides an opportunity to investigate fish and motile invertebrate response to the presence of artificial structures. Whilst this technique provides an alternative to the use of SCUBA, like all techniques for assessing marine fish assemblages it carries a level of bias that requires further quantification. Given that the majority of surveys to date on motile faunal assemblages in windfarm areas have been conducted utilising beam and otter trawl methods, future direct comparisons of stereo BRUV data with trawls would assist with further understanding of the viability of this method. Our results confirm the need for future surveys of motile fauna in these environments to utilise multiple methods [45] and to assess the fauna in close proximity to the structures rather than at a distance.

## Supporting Information

S1 Dataset Relative abundance data for 20 Stereo BRUV samples collected at different distances away from offshore wind turbines at Walney in the Irish Sea. (XLSX)
